# Dietary Sources, Bioavailability, and Functions of Ascorbic Acid (Vitamin C) and Its Role in the Common Cold, Tissue Healing, and Iron Metabolism

**DOI:** 10.7759/cureus.49308

**Published:** 2023-11-23

**Authors:** Harshit R Bhoot, Udit M Zamwar, Swarupa Chakole, Ashish Anjankar

**Affiliations:** 1 Endocrinology, Jawaharlal Nehru Medical College, Datta Meghe Institute of Higher Education and Research, Wardha, IND; 2 Community Medicine, Jawaharlal Nehru Medical College, Datta Meghe Institute of Higher Education and Research, Wardha, IND; 3 Biochemistry, Jawaharlal Nehru Medical College, Datta Meghe Institute of Higher Education and Research, Wardha, IND

**Keywords:** iron, ascorbic acid, ­wound healing, collagen synthesis, bioavailability, immunity

## Abstract

Ascorbic acid is also popularly known as vitamin C or ascorbate. It is a water-soluble vitamin. Ascorbic acid is necessary for bone formation, wound healing, connective tissue growth, and the maintenance of healthy gum tissue. Antioxidants like ascorbic acid shield the body from free radical damage. In many illnesses and conditions, vitamin C is employed as a medicinal agent. It improves the immunity of the body, reduces the severity of allergies, and aids in the management of infectious disorders. Additionally, ascorbic acid has health benefits for conditions including atherosclerosis, cancer, the common cold, iron deficiency anemia, etc. Therefore, continuous efforts may open new avenues to understand the importance of vitamin C in managing various diseases.

## Introduction and background

Vitamins are very important for numerous biological and physiological functions in the human body. Most of the vitamins cannot be made in the body, so they need to be supplemented in the diet. Ascorbic acid is a naturally occurring vitamin. Nobel laureate and Hungarian biochemist Szent-Gyorgyi made the first isolation in 1923 [1]. There are two types of ascorbic acid. They are divided into oxidized and reduced forms. Ascorbate is the reduced form, and dehydroascorbic acid is the oxidized form. They are essential antioxidants; thus, they perform a crucial role. Ascorbic acid is easily oxidized by oxygen, alkali, and high temperatures. The uronic acid pathway enables the majority of plants and animals to synthesize ascorbate from glucose or galactose. However, man and other primates can’t do this because they lack the enzyme, known as gluconolactone oxidase. This enzyme is required for the synthesis of ascorbate [2]. Ascorbic acid is essential for the synthesis and metabolism of vitamin B9 (folate) and tyrosine. Proline and glycine are also hydroxylated with its aid. It facilitates the metabolism of catecholamines, carnitine, and lysine. Additionally, ascorbic acid aids in the conversion of cholesterol to bile acids, decreasing blood cholesterol levels. As an antioxidant, ascorbic acid aids in defending the body against the negative effects of toxins, pollutants, and free radicals. One of the earliest scientists to look into the healing properties of ascorbic acid was Linus Palling. His later research on the medicinal uses of ascorbic acid was a matter of debate. The idea of using large doses of ascorbic acid, which has subsequently been widely used to treat and prevent various diseases from the common cold to cancer, was also first proposed by him [3]. Ascorbic acid aids in the treatment of diabetic and atherosclerotic patients. It is also useful in the treatment of ocular conditions like cataracts and glaucoma.

Ascorbic acid deficiency has been linked to anemia, infections, and bleeding gums. It is also associated with scurvy and poor wound healing. Ascorbic acid deficiency can result in capillary hemorrhage, muscle degeneration, atherosclerosis, neurotic disorders, etc. [3]. Ascorbic acid is often added in large doses to correct its deficiency. Toxicity is rare in comparison to fat-soluble vitamins. Ascorbic acid has also recently been studied in relation to infection and immunity. Given the wide range of biological, physiological, and therapeutic functions, this review will try to summarize some of the evidence.

## Review

Methodology

**Figure 1:**
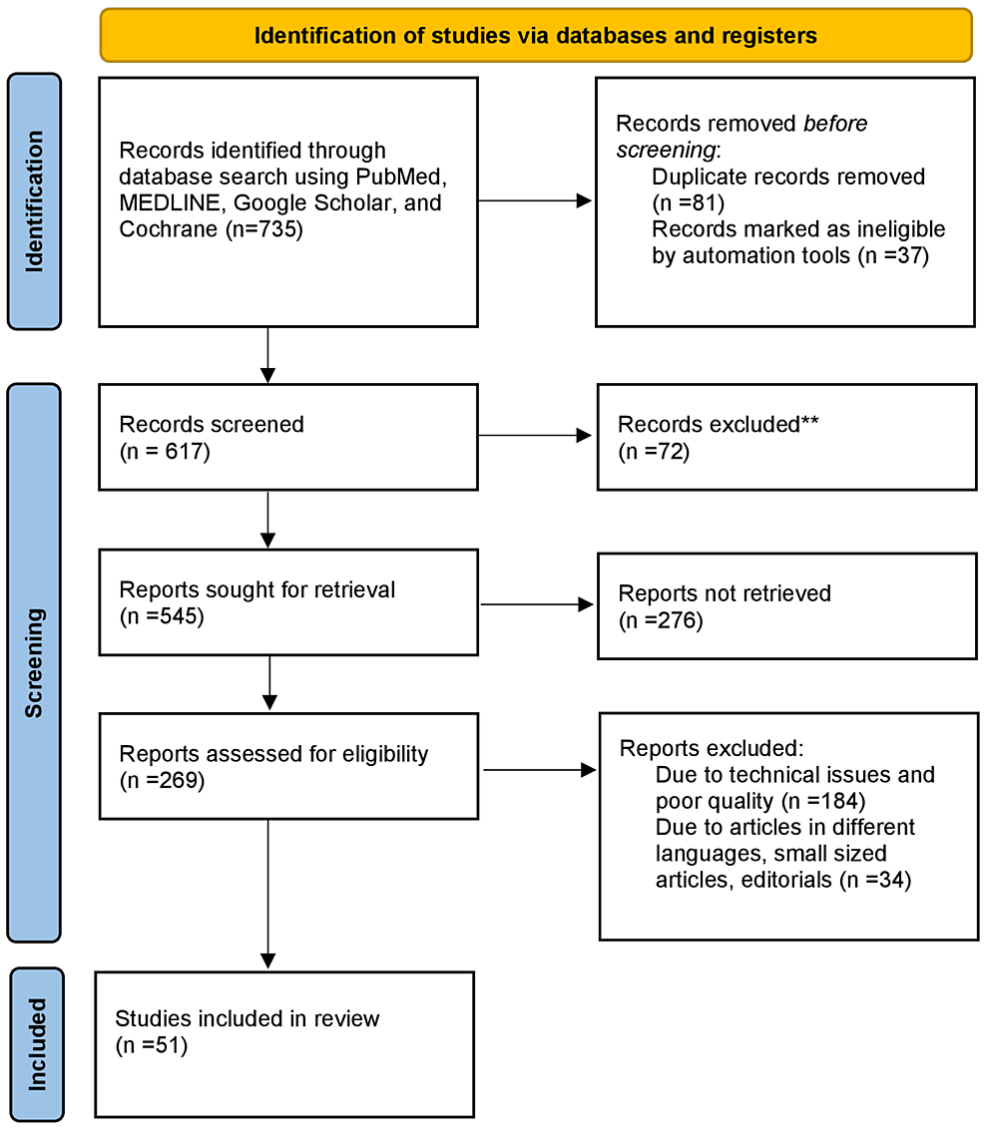
PRISMA methodology used in the study PRISMA: Preferred Reporting Items for Systematic Reviews and Meta-Analyses

The articles included in this review were taken from electronic databases PubMed, Google Scholar, and Cochrane. All the articles were in English language. The query terms were "ascorbic acid " OR "vitamin C"; "deficiency" OR "deficient”; “prevalence” OR “incidence”. This article contains studies conducted on dietary sources and bioavailability of ascorbic acid, the functions of ascorbic acid, and its role in disease prevention and cure. Articles from the past 15 years are included. Zotero software was used for the analysis of the data presented in this article. Data from a total of 51 studies has been included in this review. The Preferred Reporting Items for Systematic Reviews and Meta-Analyses (PRISMA) method used in this article is depicted in Figure 1.

Dietary sources and bioavailability

Dietary Sources

Ascorbic acid can be found in citrus fruits and vegetables like lemon, orange, strawberry, tomato, tamarind, amla, brussels sprouts, etc. Animal sources of ascorbic acid are low in content and the levels are usually less than 30-40 mg/100g. Therefore, plant sources are essential as they contain higher levels of ascorbic acid (5,000mg/100g). Passive diffusion of vitamin C in buccal cavities is the main method of absorption. However, active sodium-dependent transport of vitamin C in the gastrointestinal tract is the method of absorption by sodium-dependent vitamin C transporters (SVCT) [4,5].

Bioavailability

The bioavailability of ascorbic acid is essentially dependent on how well it is absorbed from the gut and how much of it is excreted by the kidneys. Vitamin C, whether taken with food or supplements, is digested by the small intestine by the SVCT [5-7]. Ascorbic acid is filtered through the Bowman's capsule and then reabsorbed by the transporters in the proximal convoluted tubule resulting in renal excretion of ascorbic acid [6]. Together, intestinal and renal absorption and excretion of ascorbic acid control the serum level and thus the bioavailability of ascorbic acid [8]. When vitamin C is present at low concentrations, the majority of it is digested in the small intestinal epithelium [9]. However, when vitamin C is present in high concentrations, the downregulation of SVCT1 results in a restriction of ascorbic acid absorption from both the intestine and the kidney [10,11]. This results in a physiological limit to the maximum amount of vitamin C (or its bioavailability). About 200 mmol/L is considered to be normal for healthy humans, although normal physiological serum concentrations range from 60 mmol/L to 100 mmol/L in healthy humans [12,13]. Plasma levels of vitamin C are significantly higher than circulating blood cells (platelets) due to the presence of an SVCT2 transporter in platelets, which facilitates intracellular accumulation of vitamin C [13-15].

Low levels of ascorbic acid in the body are commonly linked with stress, alcohol consumption, smoking, high fever, viral infections, antibiotic use, pain medication, exposure to CO, etc. [16]. The exact mechanism of low vitamin C levels is not well understood. It is possible that an increase in usage of ascorbic acid, or reduced absorption of vitamin C from the gut, could be the causative factors [16]. In case of excessive consumption, vitamin C and along with metabolites are eliminated through renal excretion in humans. In general, vitamin C is not toxic in humans. However, high doses of ascorbic acid can cause gastric problems [17,18]. These problems are usually mild and can be corrected with ease by decreasing the intake of ascorbic acid [18,19]. The Recommended Dietary Allowance for vitamin C is given in Table 1 [12,13].

**Table 1:** RDA for vitamin C in people of different categories and age groups RDA: Recommended Dietary Allowance

Age group	Male	Female	Pregnant women	Lactating mothers
Zero to six months	40 mg	50 mg	-	-
Seven months to one year	50 mg	50 mg	-	-
One to three years	15 mg	15 mg	-	-
Four to eight years	25 mg	25 mg	-	-
Nine to thirteen years	45 mg	45 mg	-	-
Fourteen to eighteen years	75 mg	65 mg	80 mg	115 mg
Nineteen years and above	90 mg	75 mg	85 mg	120 mg

Biochemical functions

The oxido-reduction capabilities of ascorbic acid are mediated by L-AA, a co-factor in the hydroxylation of collagen. The production of collagen and carnitine, and the metabolism of neurotransmitters, are crucial to the metabolic functioning of ascorbic acid [20]. By lowering the concentration of active metal ions, ascorbic acid speeds up this hydroxylation reaction and promotes the best possible activity of enzymes like hydroxylases and oxygenases. As a result, ascorbic acid is necessary for the formation of collagen, which is a necessary protein in the human body [21]. Ascorbic acid has been demonstrated to be involved in the synthesis and release of type 4 collagen into the culture media of experimental research [22]. Ascorbic acid 2-phosphate, a long-acting ascorbic acid derivative, has also been found to increase cell proliferation and mRNA for type 3 collagen in human osteoblasts and mesenchymal stem cells of human bone marrow [23]. Collagen, which is the main protein present in the human body, is essential for the development of numerous skin, bone, and cartilage structures, blood vessels, intervertebral discs, corneas, and eye lenses. Lack of ascorbic acid, a co-factor, has an impact on inadequate collagen production. Additionally, ascorbate is a crucial co-factor in the breakdown of carnitine generated from muscle, which is required for transferring the long-chain fatty acids to the powerhouse of the cell (mitochondria) for the production of adenosine triphosphate [24,25]. Ascorbic acid is also responsible for the conversion of neurotransmitters dopamine and norepinephrine into catecholamines, as well as catalyzing other metabolic reactions that are necessary for the highest levels of activity of hormones such as oxytocin and anti-diuretic hormone [26]. Ascorbic acid modulates the metabolic rate-limiting step of microsomal 7α-hydroxylation cholesterol in the liver, resulting in cholesterol accumulation [27]. The lack of vitamin C interferes with this conversion which causes a collection of cholesterol in the liver, resulting in hypercholesterolemia, gallstones, etc. [28-31].

Ascorbic acid and common cold

Ascorbic acid aids in preventing and relieving the common cold. This was first proposed by Pauling, who proposed a high dose of ascorbate (1-3 g) to effectively prevent the common cold [32]. Despite numerous controlled studies, the role of ascorbic acid in preventing and treating cold remains uncertain [33]. Clinical trials with various doses of ascorbic acid have demonstrated that it has no significant prophylactic effects, but does reduce the intensity and duration of common cold symptoms during the infection period. In randomized and non-randomized trials, ascorbic acid in a dose of 1g/day during the winter season did not produce the desired beneficial effects on the health of the patients in either preventive or therapeutic trials [34]. The relative beneficial effects of various ascorbic acid supplements were not identified. In studies that tested ascorbic acid following cold symptoms, increased beneficial effects with higher doses were seen as compared to the lower doses [34,35]. There has been ongoing discussion of the role of ascorbate in increasing the immune response of the body in rhinitis. Ascorbate has been demonstrated to increase the immune response by increasing T-lymphocyte proliferation. T-lymphocytes can destroy the infected targets through the production of cytokines. Additionally, ascorbic acid assists B cells in the synthesis of immunoglobulin to regulate inflammatory responses. Ascorbic acid is also responsible for blocking the pathways that cause T-cell apoptosis and thus activate or maintain the proliferation of these cells towards the infected site. This process is known to be responsible for the quick and increased reaction of the body’s immune system when the patient is suffering from rhinitis, after treatment with supplements of ascorbic acid [36,37].

Ascorbic acid and tissue healing

It is widely accepted that collagen synthesis and accumulation are essential for a wound to heal properly, followed by fiber cross-links to restore the tissue to its original tensile strength. Initial research showed that the highest tenacity of scar tissue was achieved in guinea pigs after treatment with supplements of ascorbic acid [37]. Many researches have been conducted to gain more knowledge about the role of ascorbate in wound healing and regenerative processes. An ample supply of ascorbic acid is essential for normal healing processes, particularly in postoperative patients, as ascorbic acid is rapidly utilized for collagen synthesis at the wound/burn site during the postoperative period. To expedite the healing process, it is recommended to administer 500 mg-1.0 gm of ascorbic acid daily [38]. More recently, studies have been conducted to demonstrate that early treatment with vitamin C is beneficial in the healing of wounds caused by radiation and to suggest an ascorbic acid-related treatment method to expedite wound healing in these conditions [39,40].

Ascorbic acid and iron metabolism

Ascorbic acid is known to increase the accessibility and uptake of iron (Fe) from sources other than heme iron [41]. Its supplementation has also been shown to make it easier for people to absorb iron from their food. According to a theory put forth by researchers, ascorbic acid causes iron reduction and thus promotes dietary intake of non-heme iron [42,43]. Amla, gooseberries, and other fruits high in ascorbic acid content have been found to increase the bioavailability of iron when taken as supplements. Iron bioavailability has also been hypothesized to be protective against anemia brought on by iron deficiency [44]. Additionally, new findings imply that vitamin C affects the erythropoietin receptors present in the hepG2 cells as well as inhibits the production of hepcidin [45]. As a novel regulator of the conventional transferrin Fe+ absorption pathway, ascorbate has also been proposed to work through an inherent reductive mechanism [46]. Ascorbic acid is known to display pro-oxidative activity in vitro when iron is present. This redox-active Fe may encourage the development of a hydroxyl radical, which may then cause the oxidation of lipids, DNA, or proteins [47]. Therefore, giving supplements of ascorbic acid to preterm infants with high iron levels may be detrimental because of the oxidation of the molecules [48-51]. Findings from the different studies are included in Table 2.

**Table 2:** Findings of the studies included in this article SVCT: Sodium-dependent vitamin C transporters

Author name	Year	Findings
Nishikimi et al. [2]	1994	Humans are unable to produce L-ascorbic acid due to the deficiency of this gene. This study concludes that the gene L-gulono-gamma-lactone oxidase has undergone many mutations during evolution which caused it to turn nonfunctional in humans.
Dunitz [3]	1996	Discusses the studies conducted by renowned researcher Linus Palling. His study conducted on ascorbic acid indicates that ascorbic acid should be supplemented in large doses to people for general health and prevention of diseases like the common cold and cancer.
Stevenson and Brush [4]	1969	This study was exclusively conducted on guinea pigs. This study was done to explore the transport mechanism of ascorbic acid in guinea pigs. This study concludes that the sodium-dependent vitamin C transporters function differently for different species and perform differently in humans as compared to guinea pigs.
Malo and Wilson [5]	2000	Explores the role of glucose in the transport of ascorbate in the brush border of the human small intestine. The objective was to evaluate the uptake of ascorbate and its oxidized form by the small intestine. This study concludes that the oxidized form of ascorbic acid is metabolized via facilitated diffusion whereas the transport of ascorbate is sodium-dependent and modulated by glucose.
Takanaga et al. [6]	2004	Explores the role of SVCT in ascorbate metabolism. This study concludes that there are 2 different types of SVCT for transport of ascorbate and each transporter regulates transport to different organs. They are SVCT1 and SVCT2. SVCT1 transports to the intestine, kidneys, etc. while SVCT2 transports to the eyes, lungs, etc.
Stewart and Booth [7]	1964	Discusses the bioavailability of ascorbic acid. This study concludes that the bioavailability of ascorbic acid is dependent on how much amount of it is absorbed by the gut and how much amount is excreted via the kidneys.
Ralli et al. [8]	1938	This study concludes that ascorbic acid is excreted from the kidneys by filtration and active tubular reabsorption. When ascorbic acid is absorbed at a faster rate than normal so maximum amount of it is excreted in the urine. The plasma level of ascorbate and the glomerular filtration rate are the determinants of ascorbic acid excretion.
Nelson et al. [9]	1978	This study was performed on guinea pigs and 7 normal humans. This study used the method of triple-lumen intestinal perforation to conduct the research. This article concluded that ascorbic acid supplementation acted differently in humans.
MacDonald et al. [10]	2002	Excess of ascorbic acid decreases the expression of SVCT1. This article concludes that high-dose supplementation with ascorbic acid may not be an efficient way to treat ascorbic acid deficiency.
Wilson [11]	2005	Explores the metabolism of ascorbate in the body. The two forms of ascorbic acid are metabolized via different pathways. One pathway is glucose-sensitive and the other is glucose-insensitive. This article concludes that these transport pathways are regulated under physiological conditions and altered by aging and disease.
Padayatty et al. [12]	2004	Explores the differences in plasma levels of ascorbic acid after oral and intramuscular administration. This study was conducted in an academic medical center on 17 healthy hospitalized patients. This article concludes that oral ascorbic acid does not have much significance on plasma concentrations while intramuscular ascorbic acid highly increases the plasma and urine concentrations. Intramuscular ascorbic acid may have anti-cancer effects.
Levine et al. [13]	1996	Discusses the pharmacokinetics of ascorbic acid in healthy individuals. Bioavailability of ascorbic acid, urine concentration of ascorbic acid, etc. are some of the determinants of recommended dietary allowance (RDA) for ascorbate. This article concludes ascorbate 60mg/day should be increased to 200mg/day.
Dixon and Wilson [14]	1992	Discusses the regulation of ascorbic acid transport in osteoblastic cells. This article concludes that adaptation of transport activity to substrate availability may play an important role in the physiological regulation of intracellular ascorbate levels.
Li and Schellhorn [15]	2007	Discusses the future developments and perspectives for ascorbic acid. This article concludes that plasma levels of vitamin C are significantly higher than circulating blood cells (platelets) due to the presence of an SVCT2 transporter in platelets, which facilitates intracellular accumulation of vitamin C.
Kallner et al. [16]	1979	Discusses the steady-state turnover and content of ascorbate in man. This article concludes that low levels of ascorbate in the body are commonly linked with stress, alcohol consumption, smoking, high fever, viral infections, antibiotic use, pain medication, exposure to CO, etc.
Anderson et al. [17]	1997	Explores the relationship between vitamin C and hypo and hypercholesterolemia. This study was conducted on 48 individuals who did not smoke. This article concludes that results for both low and high-cholesterol-level patients were the same.
Johnston [18]	1999	Discusses the various biomarkers for tolerable intake levels of ascorbic acid. This article concludes that high doses of ascorbate can cause gastric problems. These problems are usually mild and can be corrected with ease by decreasing the intake of ascorbic acid.
Naidu [19]	2003	Explores the role of ascorbate in human health and disease treatment from the perspective of the 21st century. This article concludes that the importance of ascorbate in the management of health is still not clear.
Levine [20]	1986	Explores the new concepts of biochemistry of vitamin C. This study concludes that the oxido-reduction capabilities of ascorbic acid are mediated by L-AA, a co-factor in the hydroxylation of collagen. The production of collagen and carnitine, and the metabolism of neurotransmitters, are crucial to the metabolic functioning of ascorbic acid.
May and Qu [21]	2005	Discusses the role of ascorbate in collagen synthesis. This study concludes that by lowering the concentration of active metal ions, ascorbic acid speeds up this hydroxylation reaction and promotes the best possible activity of enzymes like hydroxylases and oxygenases. As a result, ascorbic acid is necessary for the formation of collagen, which is a necessary protein in the human body.
Kishimoto et al. [22]	2013	Explores the role of ascorbic acid in enhancing the expression of collagen receptors and the ascorbate transporters. This article concludes that prolonged exposure to ascorbate can maintain intracellular ascorbate levels.
Maehata et al. [23]	2007	Explores the role of Type III collagen in the synthesis of osteoblastic cells. Ascorbic acid 2-phosphate, a long-acting ascorbic acid derivative, has also been found to increase cell proliferation and promote the synthesis of collagen.
Hulse et al. [24]	1978	Explores the carnitine biosynthesis and beta-hydroxylation of trimethyl lysine, and the importance of ascorbate in this process. This article concludes that the lack of ascorbic acid, a co-factor, has an impact on inadequate collagen production.
Rebouche [25]	1991	Discussed the relation between vitamin C and the synthesis of carnitine. This article concludes that ascorbate is a crucial co-factor in the breakdown of carnitine generated from muscle, which is required for transferring the long-chain fatty acids to the powerhouse of the cell (mitochondria) for the production of adenosine triphosphate (ATP).
Cameron and Pauling [26]	1973	Explores the relation between ascorbate and the glycosaminoglycans. Ascorbate is responsible for the conversion of neurotransmitters dopamine and norepinephrine into catecholamines, as well as catalyzing other metabolic reactions that are necessary for the highest levels of activity of hormones such as oxytocin and anti-diuretic hormone (ADH). This article concludes that ascorbate could be of more importance than has been generally known.
Unknown [27]	1973	Explores the catabolism of cholesterol and its relation with ascorbic acid. This study was conducted exclusively on guinea pigs. This study concludes that ascorbic acid modulates the metabolic rate-limiting step of microsomal 7α-hydroxylation cholesterol in the liver, resulting in cholesterol accumulation.
Sharma et al. [28]	1989	Discusses the increased lipid contents in guinea pigs caused by to deficiency of ascorbate. This study was conducted exclusively on guinea pigs. This article concludes that a deficiency of ascorbate causes changes in lipid contents which could promote atherogenesis.
Sharma et al. [29]	1990	Discusses the effect of ascorbate deficiency on pigs who were fed with high cholesterol diet. This article concludes that the ascorbic acid-lipid relationship has important clinical bearings and the liver could be an important site of ascorbic acid action.
Ginter et al. [30]	1982	Explores the importance of vitamin C in lipid metabolism. This article concludes that lack of ascorbate in certain reactions in the liver can disrupt fat metabolism and cause accumulation of cholesterol.
Gustafsson et al. [31]	1997	Discusses the effect of ascorbate in high doses on people with cholesterol gallstones. This study was conducted on 16 patients with gallstones. This study concludes that ascorbic acid supplementation may affect the formation of gallstones in humans.
Pauling [32]	1971	Discusses the relationship between ascorbate and cold. This article concludes that a high dose of ascorbate (1-3 g) is required to effectively prevent the common cold.
Elwood et al. [33]	1976	This study was performed to explore the role of ascorbate in the prevention of the common cold. This study was conducted on six hundred and eighty-eight women. This study concludes that despite numerous controlled studies, the role of ascorbate in the management of the common cold remains uncertain.
Hemilä and Chalker [34]	2013	Discusses the role of ascorbate in the management of the common cold. This study was conducted to find out whether ascorbate is efficient in the treatment of the common cold. This study concludes that ascorbate in a dose of 1g/day during the winter season did not produce the desired beneficial effects on the health of the patients in either preventive or therapeutic trials.
Douglas et al. [35]	1998	Discusses the use of ascorbate in case of common cold. This study concludes that supplementation of ascorbic acid following cold symptoms, increased beneficial effects with higher doses were seen as compared to the lower doses.
Campbell et al. [36]	1999	Discusses the role of ascorbate in inhibition of T cell apoptosis. This article concludes that ascorbic acid is responsible for blocking the pathways that cause T-cell apoptosis and thus activate or maintain the proliferation of these cells towards the infected site. This process is known to be responsible for the quick and increased reaction of the body’s immune system when the patient is suffering from rhinitis, after treatment with supplements of ascorbate.
Wintergerst et al. [37]	2006	Explores the relationship between ascorbate and zinc (Zn), and the positive effects on the immune system. This article concludes that ascorbate and Zn reduce the tenacity of scar tissue which was achieved in guinea pigs after treatment with supplements of ascorbate.
Bourne [38]	1946	Discusses the role of ascorbate in the healing of the wounds. An ample supply of ascorbate is essential for normal healing processes, particularly in postoperative patients, as ascorbate is rapidly utilized for collagen synthesis at the wound/burn site during the postoperative period. This article concludes that to expedite the healing process, it is recommended to administer 500 mg-1.0 gm of ascorbic acid daily.
Hellman et al. [39]	1958	Explores the metabolism and importance of ascorbic acid in man. This article concludes that ascorbate acts as an important co-factor in various reactions of the body. Hence, it is very important for the body.
Jagetia et al. [40]	2007	Explores the wound healing effects of ascorbic acid on mice and compares it with humans. This article concludes that treatment with vitamin C is beneficial in the healing of wounds caused by radiation and suggests an ascorbic acid-related treatment method to expedite wound healing in these conditions.
Hallberg [41]	1981	Explores the role of ascorbate in increasing the bioavailability of dietary Fe in man. Ascorbate is known to increase the accessibility and uptake of iron (Fe) from sources other than heme iron. This article concludes that ascorbate increases the bioavailability of iron.
Bendich and Cohen [42]	1990	Discusses the importance of ascorbate in the absorption of iron. This article concludes that ascorbic acid causes iron reduction and thus promotes dietary intake of non-heme iron.
Zhang et al. [43]	2013	Explores the effects of dietary factors on the metabolism of Fe after ascorbate supplementation. This article concludes that people should consume Fe-fortified foods.
Gowri et al. [44]	2001	Discusses the effects on the health of the body after consumption of ascorbate-rich fruits. This study concludes that Amla and other fruits high in ascorbic acid content have been found to increase the bioavailability of Fe when taken as supplements.
Chiu et al. [45]	2012	Discusses the effects of ascorbate on the functioning of receptors involved in blood formation. This article concludes that vitamin C affects the erythropoietin receptors present in the hepG2 cells as well as inhibits the production of hepcidin.
Lane et al. [46]	2013	Explores the effects of ascorbate on the uptake of transferrin Fe. This article concludes that as a novel regulator of the conventional transferrin Fe+ absorption pathway, ascorbate has also been proposed to work through an inherent reductive mechanism.
Samuni et al. [47]	1983	Discusses the cytotoxicity of ascorbate and metallic ions. This article concludes that ascorbic acid displays pro-oxidative activity in vitro when iron is present.
Minetti et al. [48]	1992	Discusses the effects of Fe on oxidation of ascorbic acid. This article concludes that the redox-active Fe may encourage the development of a hydroxyl radical, which may then cause the oxidation of lipids, DNA, or proteins.
Berger et al. [49]	1995	Discusses the effects of ascorbate supplementation in preterm and term infants with high iron content. This article concludes that giving supplements of ascorbic acid to preterm infants with high iron levels may be detrimental because of the oxidation of the molecules.
Halliwell [50]	1996	Discusses whether ascorbate is an antioxidant or pro-oxidant in vivo. This study concludes that it is still a question whether these pro-oxidant properties of ascorbate are of any importance.
Herbert et al. [51]	1996	Discusses the ascorbic acid-driven free radical generation from iron. This article concludes that ascorbate supplementation can have an impact on the health of infants.

## Conclusions

Ascorbic acid is a very necessary nutrient. It can be naturally gained from sour fruits and vegetables like lemon, orange, tamarind, amla, brussels sprouts, etc. It plays an essential role in bone development, wound healing, etc. Vitamin C deficiency can cause scurvy and bone deformities. It acts as an important factor in the synthesis of collagen and is also very necessary for the conversion of neurotransmitters dopamine and norepinephrine into catecholamines. Ascorbate aids in the treatment and prevention of a disease like the common cold and is essential for tissue healing repair, and maintenance of the metabolism of iron in the body. To conclude, ascorbic acid is an extremely necessary nutrient for the body and people should regularly keep a check on their vitamin C levels and look for any possible symptoms. People should include ample amounts of fruits and vegetables rich in ascorbic acid in their daily diet to keep their ascorbic acid levels in check as prevention is always better than cure.
